# The correlation between KRAS and TP53 gene mutations and early growth of pulmonary nodules

**DOI:** 10.1186/s13019-024-02927-0

**Published:** 2024-06-26

**Authors:** Bin Zhao, Bin Li, Haoxin Guo, Qingtao Zhao, Xiaopeng Zhang, Huanfen Zhao, Wenfei Xue, Wei Li, Guochen Duan, Shun Xu

**Affiliations:** 1 Department of Thoracic Surgery, Hebei General Hospital, Shijiazhuang, 050057 People’s Republic of China; 2 Graduate School, Hebei Medical University, Shijiazhuang, 050011 People’s Republic of China; 3 Hebei Bio-High Technology Development CO., LTD, Shijiazhuang, 050011 People’s Republic of China; 4 Department of Thoracic Surgery, Children’s Hospital of Hebei Province, Shijiazhuang, 050000 People’s Republic of China; 5 Department of Thoracic Surgery, The First Hospital of China Medical University, Shenyang, 110000 People’s Republic of China

**Keywords:** KRAS, TP53, Early pulmonary nodules, Tumor growth

## Abstract

**Purpose:**

The purpose of this study is to investigate whether gene mutations can lead to the growth of malignant pulmonary nodules.

**Methods:**

Retrospective analysis was conducted on patients with pulmonary nodules at Hebei Provincial People’s Hospital, collecting basic clinical information such as gender, age, BMI, and hematological indicators. According to the inclusion and exclusion criteria, 85 patients with malignant pulmonary nodules were selected for screening, and gene mutation testing was performed on all patient tissues to explore the relationship between gene mutations and the growth of malignant pulmonary nodules.

**Results:**

There is a correlation between KRAS and TP53 gene mutations and the growth of pulmonary nodules (*P* < 0.05), while there is a correlation between KRAS and TP53 gene mutations and the growth of pulmonary nodules in the subgroup of invasive malignant pulmonary nodules (*P* < 0.05).

**Conclusion:**

Mutations in the TP53 gene can lead to the growth of malignant pulmonary nodules and are correlated with the degree of invasion of malignant pulmonary nodules.

**Supplementary Information:**

The online version contains supplementary material available at 10.1186/s13019-024-02927-0.

## Introduction

In the world, due to the continuous development of society, air and environmental pollution problems deeply affect people’s lives and health, especially the incidence of respiratory diseases is increasing [1]. Among them, malignant lung nodules are the most serious respiratory diseases, and the incidence and mortality rate are increasing year by year [2]. Most malignant pulmonary nodules do not have clear symptoms in the early stages of onset, but during this period, malignant pulmonary nodules already exist in the human body [3]. In order to maintain people’s health and life safety, early diagnosis and screening of malignant pulmonary nodules are particularly important. Mastering the timing and treatment methods of diagnosis and treatment for pulmonary malignant nodules is of great significance in controlling the mortality rate of pulmonary malignant nodules.

Nowadays, chest spiral CT has become an important detection method for early screening of pulmonary nodules. Whether and to what extent the nodules displayed on chest CT grow directly determines the overall treatment strategy of patients with pulmonary nodules [4]. In recent years, genetic research on lung tumors has become a hot topic. The development of drugs targeting various gene loci has also promoted the development of targeted therapy for tumors. However, there is little clinical research discussing the association between gene mutations and the growth of early pulmonary nodules. This article aims to explore the correlation between gene mutations and nodule growth through clinical research, in order to find more accurate methods for diagnosing early pulmonary nodules and better develop diagnosis and treatment strategies for patients.

## Methods

### Basic information

We collected 150 patients with pulmonary nodules who were treated in the Department of Thoracic Surgery of Hebei Provincial People’s Hospital from January 2021 to March 2023 and underwent genetic mutation testing, and screened according to inclusion and exclusion criteria. Finally, 85 patients who met the requirements were retained. We collected clinical information such as gender, age, BNI, and hematological indicators from these patients, and performed grouping analysis on the patients.

### Inclusion and exclusion criteria

#### Inclusion criteria

(1) Early stage pulmonary nodule patients treated at Hebei Provincial People’s Hospital; (2) In the stable group, preoperative chest CT was performed at least 3 months apart, while in the growth group, patients had an increase in nodule diameter or a significant increase in solid composition; (3) All patients were treated for the first time and had not received anti-tumor treatment such as chemotherapy or radiotherapy before admission; (4) Complete clinical data; (5) The results of genetic testing are reliable and effective.

#### Exclusion criteria

(1) Patients without two CT results or with an interval of less than three months between two CT tests; (2) Complicated with other chest diseases such as pleural effusion and pneumothorax, resulting in CT affecting patients who cannot accurately measure the diameter of nodules; (3) Patients with missing clinical data; (4) Patients with benign lesions confirmed by pathological examination.

### Grouping standard

1. Growth group: At least two deputy chief physicians of the imaging department looked at CT and confirmed that the diameter of the nodule increased by ≥ 3 mm or the diameter of the tumor increased by at least 20%, and the solid component increased significantly.
2. Stable group: preoperative chest CT examination, postoperative observation or measurement of the actual diameter of the nodule after surgical resection, and then re-examination of CT, it was found that the nodule had no growth or no increase in solid components, or no increase in the volume and quality of the nodule.

### Processing of CT images

The bronchial CT images of the patient were scanned by a 128-slice CT machine from Siemens Definition Flash (Siemens AG, Germany). Scanning parameters: tube voltage 120 kV, tube current 200 ~ 300 mA, scanning layer thickness 2 mm, layer spacing 1 mm. The growth of the nodule is done and tested by at least two deputy chief physicians of the imaging department.

### Gene mutation detection methods

1. Paraffin sections were stained for HE staining, and the proportion of tumor cells was evaluated by microscopic examination, and > 5% was selected as qualified samples;
2. Refer to the instructions of the FFPE sample nucleic acid extraction kit (Biospin paraffin-embedded tissue genomic DNA extraction kit or E.Z.N.A. FFPE tissue RNA extraction kit) for nucleic acid extraction; The brief description is as follows:
  - Add the deparaffinization solution to the tissue to be examined, centrifuge (14,000 g, 2 min) after incubation to remove the upper paraffin.
  - Lysis and digestion of the remaining tissue fraction (more than 60 min).
  - The digested samples were combined, washed, and eluted, and nucleic acids were collected.
3. The extracted DNA/RNA nucleic acids were quantified using NanoDrop;
4. Prepare PCR reaction solution according to the requirements of the instruction manual of the detection kit (IntelliPlex™ Lung Cancer Panel-DNA &RNA);
5. After the detection reagent mixture is divided into the PCR tube, the sample to be tested is added;
6. DNA and RNA were set with different temperature programs in the amplification instrument for PCR amplification;
7. Quantitative analysis and signal statistics were carried out on the amplification products after the amplification was completed, and the detection results were obtained.

### Statistical analysis

The data analysis was conducted using SPSS26.0 statistical software. Wilcoxon rank sum test was used to analyze the correlation between all gene site mutations involved in gene testing results and early lung nodule growth. Binary logistic regression analysis was used to verify the relationship between gene mutations and lung nodule growth. Subsequently, patients were grouped based on wettability and non-wettability for subgroup analysis to explore whether there was a correlation between gene mutations and lung nodule growth. *P* < 0.05 indicates that the difference is statistically significant.

## Results

### Basic data analysis

85 patients were included in the study, of which 40 (47.1%) were male, with a median age of 63 years (interquartile range, IQR = 13.5), and 32 (37.6%) patients had nodules located in the left lung. According to clinical data, there were 80 (94.1%) patients with lung adenocarcinoma, and the rest were patients with lung squamous cell carcinoma. 71 patients (83.5%) with clinical staging earlier than stage II, and a total of 44 patients (51.8%) with invasive lung cancer. The detected mutated genes include EGFR, ALK, KRAS, PIK3CA, c-MET, TP53, CTNNB1, and NF1. The number of patients with mutations in each genotype was 47 (55.3%), 1 (1.2%), 14 (16.5%), 6 (7.1%), 2 (2.4%), 21 (24.7%), 1 (1.2%), and 2 (2.4%). The imaging manifestations of patients were also statistically analyzed, with a total of 13 patients (15.3%) exhibiting lobular sign, 17 patients (20.0%) exhibiting spicule sign, and 5 patients (5.9%) exhibiting vascular sign. The median of various hematological indicators is as follows: Alb is 41.90, WBC is 6.14, N is 3.90, L is 1.68, M is 0.31, Hb is 135, and PLT is 226 (Table 1).

**Table 1:** Basic clinical data analysis

		*n* (%) or median (IQR)
Age(y)		63(13.5)
BMI		25.46(4.51)
Gender	Male	40(47.1)
	Female	45(52.9)
The location of the nodule	Left	32(37.6)
	Right	53(62.4)
Clinical stages	≤ Stage II	71(83.5)
	> Stage II	14(16.5)
Pathological type	Adenocarcinoma	80(94.1)
	Other	5(5.9)
Degree of tumor invasion	Infiltrative	44(51.8)
	Non invasive	41(48.2)
Lobulation sign	Negative	72(84.7)
	Positive	13(15.3)
Spiculation sign	Negative	68(80.0)
	Positive	17(20.0)
Vascular sign	Negative	80(94.1)
	Positive	5(5.9)
Nodular growth	Grown	54(63.5)
	Ungrown	31(36.5)
EGFR	Wild type	38(44.7)
	Mutant type	47(55.3)
ALK	Wild type	84(98.8)
	Mutant type	1(1.2)
KRAS	Wild type	71(83.5)
	Mutant type	14(16.5)
PIK3CA	Wild type	79(92.9)
	Mutant type	6(7.1)
c-MET	Wild type	83(97.6)
	Mutant type	2(2.4)
TP53	Wild type	64(75.3)
	Mutant type	21(24.7)
CTNNB1	Wild type	84(98.8)
	Mutant type	1(1.2)
NF1	Wild type	83(97.6)
	Mutant type	2(2.4)
Alb		41.9
WBC		6.14
N		3.90
L		1.68
M		0.31
Hb		135
PLT		226

n: number of patients; IQR: interquartile range

After descriptive analysis of basic clinical information, we grouped all patients according to whether the nodules grew or not, and statistically analyzed clinical observation indicators including gender, age, BMI, nodule site, clinical stage, pathological type, invasion degree, Alb, WBC, N, L, M, Hb, PLT, lobulation sign, hair prick sign, and vascular sign, explore whether these indicators are related to nodule growth through Wilcoxon rank sum test. According to the analysis results, only BMI showed intergroup differences between the growth group and the stable group (*P* = 0.013), indicating a correlation between BMI and nodule growth (Table 2).

**Table 2:** Correlation analysis between clinical data and nodule growth

	Statistic W	Progressive significance(*P*)
Gender	1180.5	0.107
Age	1262	0.516
BMI	2050	0.013
The location of the nodule	1319	0.879
Clinical stages	1286	0.504
Pathological type	1298	0.433
Degree of tumor invasion	1288.5	0.639
Alb	1317.5	0.887
WBC	2314	0.942
N	1288	0.681
L	2231	0.406
M	2220.5	0.354
Hb	1254.5	0.473
PLT	2142.5	0.101
Lobulation sign	1259	0.279
Spiculation sign	2288	0.654
Vascular sign	1298	0.433

Next, we conducted statistical analysis on the results of gene testing. We still group patients based on nodule growth and analyze whether mutations at all gene loci are correlated with nodule growth. The final statistical results showed that there were intergroup differences in KRAS and TP53 (*P* < 0.05) (Table 3).

**Table 3:** Correlation analysis between gene testing results and nodule growth

	Statistic W	Progressive significance(*P*)
EGFR	1199.5	0.157
ALK	2295	0.187
KRAS	2114	0.003
PIK3CA	1282.5	0.299
c-MET	1302	0.281
TP53	1135	0.016
CTNNB1	2295	0.187
NF1	1302	0.281

Based on the above research results, we have reason to believe that KRAS and TP53 are correlated with nodule growth. Therefore, we used BMI and these two gene loci as observation indicators and conducted binary logistic regression analysis on their data results. The results showed that KRAS and TP53 had a significant correlation with nodule growth (*P* < 0.05), while BMI no longer showed statistical significance with nodule growth (*P* > 0.05) (Table 4).

**Table 4:** Binary logistic analysis of BMI, KRAS, and TP53 with nodule growth

	Standard Error	Significance	Exp(B)	95% confidence interval for EXP(B)	
				Lower	Upper
BMI	0.081	0.056	0.856	0.731	1.004
KRAS	0.751	0.012	0.151	0.035	0.657
TP53	0.816	0.014	7.396	1.495	36.6

Subsequently, we grouped all patients based on nodule infiltration and conducted subgroup analysis of patient data. Through binary logistic regression analysis, we found that in the infiltration group, the growth of nodules remained closely correlated with KRAS and TP53 (*P* < 0.05) (Table 5).

**Table 5:** Binary logistic analysis of KRAS and TP53 in infiltration group and nodule growth

	Standard Error	Significance	Exp(B)	95% confidence interval for EXP(B)	
				Lower	Upper
KRAS	1.140	0.014	0.061	0.007	0.568
TP53	1.391	0.026	22.148	1.451	338.071

## Discussion

In terms of current clinical research content, the main trend in predicting the growth of pulmonary nodules still relies on imaging examination methods mainly using low-dose spiral CT [5, 6]. In addition, studies have also explored the factors that affect the growth of pulmonary nodules by analyzing gene expression pathways [7–9]. This study is based on existing research and combined with collected patient data, aiming to analyze and explore the genotypes that affect the growth of pulmonary nodules at the clinical level, in order to more accurately predict the development trend of nodules in the screening process of pulmonary nodules, and thus find more suitable clinical intervention opportunities to achieve better treatment effects.

Firstly, we conducted a statistical analysis of the patient’s basic clinical information, and the results showed a correlation between the patient’s BMI and nodule growth (*P* = 0.013). As is well known, BMI represents the relationship between patient body mass and height, and its clinical significance lies in reflecting the patient’s nutritional and energy status. It has been proved that BMI is related to the occurrence, development and prognosis of breast cancer, rectal cancer, lung cancer, pancreatic cancer and other solid tumors [10–13]. Based on our analysis results, we have reason to believe that BMI is to some extent related to nodule growth. The inherent correlation may be due to the need for the human body to provide basic energy and nutrients to tumor cells during the process of tumor growth. However, in the subsequent binary logistic regression analysis, the correlation between BMI and nodule growth was not statistically significant, indicating that BMI cannot be an independent predictor of nodule growth, and its regulation of pulmonary nodule growth is influenced by other factors.

Subsequently, we conducted statistical analysis on the gene testing results, and the results showed intergroup differences between KRAS (*P* = 0.003) and TP53 (*P* = 0.016), indicating a correlation between KRAS and TP53 and nodule growth. The KRAS gene belongs to the RAS gene family, among which KRAS is the most common mutation in human cancer. In lung cancer patients, KRAS gene mutations mainly affect lung adenocarcinoma [14]. In the early stage of lung adenocarcinoma, mutations in the KRAS gene have a significant impact, especially on the premise of ADC, which is atypical adenomatous hyperplasia (AAH) [15]. Due to the fact that the patients we selected were all early-stage lung tumor patients, the relationship between KRAS and nodule growth was also more evident in this study. It is reported that in breast cancer patients, low level of KRAS expression can induce cell proliferation and breast epithelial hyperplasia, while high level of KRAS expression can cause cell senescence [16]. However, there are also reports suggesting that the widespread expression of KRAS during embryonic development can induce precancerous epithelial hyperplasia in the lung and gastrointestinal tracts [17]. In our study, we found a correlation between KRAS mutations and nodule growth, and in subsequent analysis, binary logistic analysis confirmed that KRAS can serve as an independent predictor of pulmonary nodule growth (*P* = 0.012). This means that the expression of KRAS can promote the growth of lung nodules in the population of patients with early lung cancer, namely lung nodules.

The TP53 gene is located on chromosome 17 and is a tumor suppressor gene [18]. The mutation of TP53 can be reflected in most malignant tumors and can be seen in all types of lung cancer [19, 20]. Previous literature has shown that mutations in TP53 can activate abnormal cell proliferation, thereby promoting tumor growth. Its main mechanism of action is to regulate the autophagy process of tumors, allowing autophagosomes within cells to phagocytose organelles or proteins and fuse with lysosomes. This process can promote tumor cells’ resistance to harsh microenvironments, thereby increasing their resistance to radiotherapy, chemotherapy, or targeted therapy [21, 22]. But the autophagy process can inhibit the growth and reproduction of abnormal cells such as tumors in the absence of tumor occurrence [23]. Therefore, the specific role of TP53 in tumor growth is also worth exploring. Due to the frequent occurrence of TP53 as a concomitant mutation, relevant studies have explored the prognosis and drug resistance of TP53 and other genotype co mutations [24–27]. In this study, Wilcoxon rank sum test analysis showed a correlation between TP53 and nodule growth (*P* = 0.016), indicating that TP53 can lead to the growth of early pulmonary nodules. Moreover, binary logistic regression analysis also confirmed that TP53 can serve as an independent predictor of early lung nodule growth (*P* = 0.014).

This study further divided all patients into invasive and non-invasive groups based on their degree of nodule invasion, and explored whether KRAS and TP53 mutations are correlated with nodule growth in both subgroups. The results showed that there was still a correlation between KRAS and TP53 mutations and early lung nodule growth in the infiltrating group (*P* < 0.05), but there was no longer a correlation in the non-infiltrating group (*P* > 0.05). This indicates that KRAS and TP53 mutations are more closely associated with tumor growth in cases of strong tumor invasiveness.

From the perspective of the patient population included in this study, the majority (83.5%) of patients with early pulmonary nodules with clinical staging earlier than stage II. According to the guidelines, there are surgical opportunities for such patients. In clinical practice, most patients undergoing physical examination cannot predict the growth trend of pulmonary nodules solely based on one CT examination. Once the visit is delayed, it will greatly increase the difficulty of treatment and reduce the level of prognosis. Therefore, after the patient first discovers pulmonary nodules, genetic testing can be used to evaluate the mutation status of KRAS and TP53. If a patient experiences KRAS or TP53 mutations, the risk of nodular enlargement is higher. In this case, it is recommended that patients intervene with pulmonary nodules as soon as possible to achieve better treatment outcomes.

There are still some limitations in this study: (1) This is a single center, retrospective study that may lead to selection bias; (2) The overall sample size is relatively small, and further expansion of the sample size is needed to verify the results; (3) This study only considered the increase in lung nodule volume, and did not include patients with increased nodule density, which may also affect the final results; (4) This study only conducted genetic testing on existing common genotypes, and further research will be conducted in the future to find more gene mutation types with increased imaging nodules. KRAS and TP53, as gene loci for predicting the growth of pulmonary nodules, still need to further explore their specific regulatory mechanisms, and multicenter and prospective clinical studies are needed to validate their value.

## Conclusion

(1) Mutations in KRAS and TP53 are associated with the growth of pulmonary nodules, and both are independent predictors of pulmonary nodule growth; (2) The KRAS and TP53 mutations are more significantly associated with the growth of invasive lung cancer. These results provide theoretical guidance for formulating treatment plans for patients, and also provide research ideas for further exploring the influencing factors of early lung cancer growth.

### Electronic supplementary material

Below is the link to the electronic supplementary material.

Supplementary Material 1

## Data Availability

No datasets were generated or analysed during the current study.
