# Supplementary material for: The correlation between KRAS and TP53 gene mutations and early growth of pulmonary nodules
Source: J Cardiothorac Surg. 2024 Jun 26;19:376. doi: 10.1186/s13019-024-02927-0 (PMC11200870; doi:10.1186/s13019-024-02927-0)
Supplement: Supplementary file 1 — Supplementary Material 1 [file 13019_2024_2927_MOESM1_ESM.pdf]

Hebei General Hospital

Ethics Committee Application for Approval of Research Protocol

NO. 2023126

Paper Title: The Correlation between KRAS and TP53 Gene Mutations and Early Growth of Pulmonary Nodules

Article author: Bin Zhao, Bin Li, Haoxin Guo, Qingtao Zhao, Xiaopeng Zhang, Huanfen Zhao, Wenfei Xue, Wei Li, Guochen Duan

**Documents for Application**	■ Application form<br>■ the thesis
**Consequence**	**After reviewing, this paper conforms to the principals of medical ethics. It is accepted to publish.**

Committee Leader（Signature）:

Hebei General Hospital Ethics Committee

Date: Sept. 28 2023

Address: NO.348 Heping West Road Shijiazhuang City, Hebei Prov. CHINA
TEL: 0311-85988311
